# HLA-DQB1*03 Confers Susceptibility to Chronic Hepatitis C in Japanese: A Genome-Wide Association Study

**DOI:** 10.1371/journal.pone.0084226

**Published:** 2013-12-20

**Authors:** Daiki Miki, Hidenori Ochi, Atsushi Takahashi, C. Nelson Hayes, Yuji Urabe, Hiromi Abe, Tomokazu Kawaoka, Masataka Tsuge, Nobuhiko Hiraga, Michio Imamura, Yoshiiku Kawakami, Hiroshi Aikata, Shoichi Takahashi, Norio Akuta, Fumitaka Suzuki, Kenji Ikeda, Hiromitsu Kumada, Yoshiyasu Karino, Joji Toyota, Tatsuhiko Tsunoda, Michiaki Kubo, Naoyuki Kamatani, Yusuke Nakamura, Kazuaki Chayama

**Affiliations:** 1 Laboratory for Digestive Diseases, Center for Integrative Medical Sciences, RIKEN, Hiroshima, Japan; 2 Department of Gastroenterology and Metabolism, Applied Life Sciences, Institute of Biomedical & Health Sciences, Hiroshima University, Hiroshima, Japan; 3 Liver Research Project Center, Hiroshima University, Hiroshima, Japan; 4 Laboratory for Statistical Analysis, Center for Integrative Medical Sciences, RIKEN, Yokohama, Japan; 5 Natural Science Center for Basic Research and Development, Hiroshima University, Hiroshima, Japan; 6 Department of Hepatology, Toranomon Hospital, Tokyo, Japan; 7 Department of Gastroenterology, Sapporo Kosei General Hospital, Hokkaido, Japan; 8 Laboratory for Medical Science Mathematics, Center for Integrative Medical Sciences, RIKEN, Yokohama, Japan; 9 Laboratory for Genotyping Development, Center for Integrative Medical Sciences, RIKEN, Yokohama, Japan; 10 Laboratory of Molecular Medicine, Human Genome Center, The Institute of Medical Science, University of Tokyo, Tokyo, Japan; National Cancer Institute, National Institutes of Health, United States of America

## Abstract

Hepatitis C virus (HCV) establishes a chronic infection in 70-80% of infected individuals. Many researchers have examined the effect of human leukocyte antigen (HLA) on viral persistence because of its critical role in the immune response against exposure to HCV, but almost all studies have proven to be inconclusive. To identify genetic risk factors for chronic HCV infection, we analyzed 458,207 single nucleotide polymorphisms (SNPs) in 481 chronic HCV patients and 2,963 controls in a Japanese cohort. Next, we performed a replication study with an independent panel of 4,358 cases and 1,114 controls. We further confirmed the association in 1,379 cases and 25,817 controls. In the GWAS phase, we found 17 SNPs that showed suggestive association (*P* < 1 × 10^-5^). After the first replication study, we found one intronic SNP in the *HLA-DQ* locus associated with chronic HCV infection, and when we combined the two studies, the association reached the level of genome-wide significance. In the second replication study, we again confirmed the association (*P*_combined_ = 3.59 × 10^−16^, odds ratio [OR] = 0.79). Subsequent analysis revealed another SNP, rs1130380, with a stronger association (OR=0.72). This nucleotide substitution causes an amino acid substitution (R55P) in the HLA-DQB1 protein specific to the DQB1*03 allele, which is common worldwide. In addition, we confirmed an association with the previously reported *IFNL3-IFNL4* locus and propose that the effect of DQB1*03 on HCV persistence might be affected by the *IFNL4* polymorphism. Our findings suggest that a common amino acid substitution in HLA-DQB1 affects susceptibility to chronic infection with HCV in the Japanese population and may not be independent of the *IFNL4* genotype.

## Introduction

At least 1.5 million people in Japan and more than 200 million people worldwide are chronically infected with the hepatitis C virus (HCV) [1, 2]. In many western countries and Japan, HCV infection is the most common risk factor for hepatocellular carcinoma (HCC) [3, 4]. HCV establishes a chronic infection in approximately 70-80% of infected individuals [5, 6], and host genetic factors in addition to viral factors are assumed to partially explain the heterogeneity in HCV persistence or clearance. Many researchers have examined the effect of human leukocyte antigen (HLA) on viral persistence because of its critical role in the immune response to HCV [7-10] and because numerous HLA class II alleles have been reported to affect HCV clearance; however, results of almost all such studies have been inconclusive. On the other hand, a strong association between HCV clearance and variants close to interferon-λ3 (*IFNL3*), previously known as *IL28B*, has been consistently replicated in multiple studies, including two genome-wide association studies (GWAS). Those studies have been performed mainly using individuals of European and African, but not Asian, ancestry [11-13]. Recently it was reported that a dinucleotide polymorphism (ss469415590) creates or disrupts an open reading frame belonging to a newly discovered gene, *IFNL4* [14]. It is well known that there are great differences among ethnic groups in allele frequencies in both HLA class II and *IFNL4* variants or variants from the IFNL3-IFNL4 region. To identify genetic markers associated with chronic HCV infection in the Japanese population, we conducted a case control study consisting of a GWAS and two replication studies that included 6,218 cases with chronic HCV infection and 29,894 controls for a total of 36,112 Japanese individuals. In Japan, the prevalence of chronic hepatitis C is approximately 1%, and it was reported that the frequency of individuals testing positive for antibody to HCV (anti-HCV) was 0.49% among 3,485,648 Japanese first-time blood donors [15]. As the risk of exposure to HCV is low in Japan and the potential occurrence of HCV infection in controls is unlikely to affect the results, we tried to detect loci involved in susceptibility to HCV persistence using a large number of individuals in the general population as controls.

## Results

### GWAS and Replication Studies

In the GWAS phase, single nucleotide polymorphism (SNP) genotyping was performed using the Illumina HumanHap610-Quad BeadChip for cases and the Illumina HumanHap550v3 BeadChip for controls. Genotype concordance between these two BeadChips was 99.99% among 182 duplicated samples, indicating the low possibility of genotyping error. 458,207 SNPs passed quality control filters and were analyzed using an additive model for genotype-phenotype association in 481 patients with chronic HCV infection and 2,963 controls. Principal component analysis revealed no population substructure in our population. In addition, a quantile-quantile plot using the results of the Cochran-Armitage trend test showed that the inflation factor, λ, was 1.007, indicating a low probability of false-positive associations resulting from population stratification (Figure S1A). Using the additive model, one SNP reached the genome-wide significance level for association after Bonferroni correction (calculated as *P* < 0.05/458,207 = *P* < 1.09 x 10^-7^), and another 24 SNPs showed suggestive association (*P* < 1 x 10^-5^) in our GWAS (Figure S1B and Table S1). Next, we conducted the first replication study to validate the results of the GWAS phase using 4,358 cases and 1,114 controls. We performed genotyping of 18 SNPs with *P* values < 1x10^-5^ in the GWAS phase, after excluding 7 SNPs with *r* ^2^ = 1 in the same locus based on the HapMap-JPT database, and successfully genotyped all SNPs using multiplex PCR-based Invader assay (Table S2), except for rs9275563, which is moderately linked with rs9275572 (*r* ^2^ = 0.56). Only rs9275572 in the HLA-DQ locus on chromosome 6 showed significant replication of the association (*P* < 0.05/17), and the association reached a genome-wide significance level when we combined the two studies using the Mantel-Haenszel method (*P* = 2.04 × 10^−12^, odds ratio [OR] = 0.75; Table 1). We further confirmed the association in 1,379 cases and 25,817 controls and found a highly significant association with chronic HCV after meta-analysis of all three studies (*P*_combined_ = 3.59 × 10^−16^, OR = 0.79, 95% confidence interval [CI] 0.75-0.84). As shown in Table 1, we observed no heterogeneity among the three studies (heterogeneity test *P* = 0.113).

**Table 1:** Summary of GWAS and replication studies.

SNP		Case				Control				MAF					
*locus*	Study	TT	TC	CC		TT	TC	CC		Case	Control	OR	(95%CI)^a^	*P* ^b^	*P*_het_^c^
rs9275572	GWAS	96	217	168		366	1317	1280		0.425	0.346	0.71	(0.62-0.82)	2.62E-06	
*HLA-DQB1*	1st replication	634	2049	1673		119	474	519		0.381	0.320	0.77	(0.69-0.85)	1.40E-07	
	2nd replication	206	695	477		3279	11284	10260		0.402	0.359	0.84	(0.77-0.90)	7.63E-06	
	Combined GWAS and 1st replication study^d^											0.75	(0.69-0.81)	2.04E-12	4.25E-01
	Combined all studies^e^											0.79	(0.75-0.84)	3.59E-16	1.13E-01

MAF; minor allele frequency, OR; odds ratio, CI; confidence interval.

Odds ratios and *P* values for independence test were calculated by the Mantel-Haenszel method.

^a^Odds ratios of protective allele (C) from two-by-two allele frequency table. ^b^ *P* value of Cochran-Armitage trend test.

^c^Result of Breslow-Day test. ^d^Meta-analysis of GWAS and 1st replication study. ^e^Meta-analysis of all three studies.

### Multiple Logistic Regression Analysis and Stratified Analysis

We next evaluated the association in more detail using the first replication set. Both HCV case and healthy control samples were collected at Hiroshima University. After adjusting for gender and age using multiple logistic regression analysis, the rs9275572 C allele remained highly significant with an OR = 0.79 (95% CI 0.70-0.89) (Table S3). Our study also confirmed a stronger protective effect of female gender compared to male gender [12, 16]. Subsequently, we analyzed the effect of rs9275572 according to gender and age. As shown in Table S4A, we observed no significant difference between subgroups. In addition to host genetic factors, such as *HLA* alleles, the distribution of viral genotypes is also highly variable around the world, and it has been suggested that different HCV genotypes result in different clinical outcomes [17, 18]. For example, HCV genotype 1, the most common genotype in Japan, shows stronger resistance against IFN-based anti-viral therapy than HCV genotype 2, the second most frequently encountered genotype in Japan [19, 20]. Hence, we stratified HCV cases by HCV genotype and compared each with healthy controls. We observed that the effect tended to be slightly greater in genotype 1 (OR = 0.74 [0.66-0.82]) than genotype 2 (OR = 0.83 [0.74-0.94]), but there was no significant difference (heterogeneity test *P* = 0.127) (Table S4B).

### Detailed Analysis of HLA-DQ alleles

**Figure 1:**
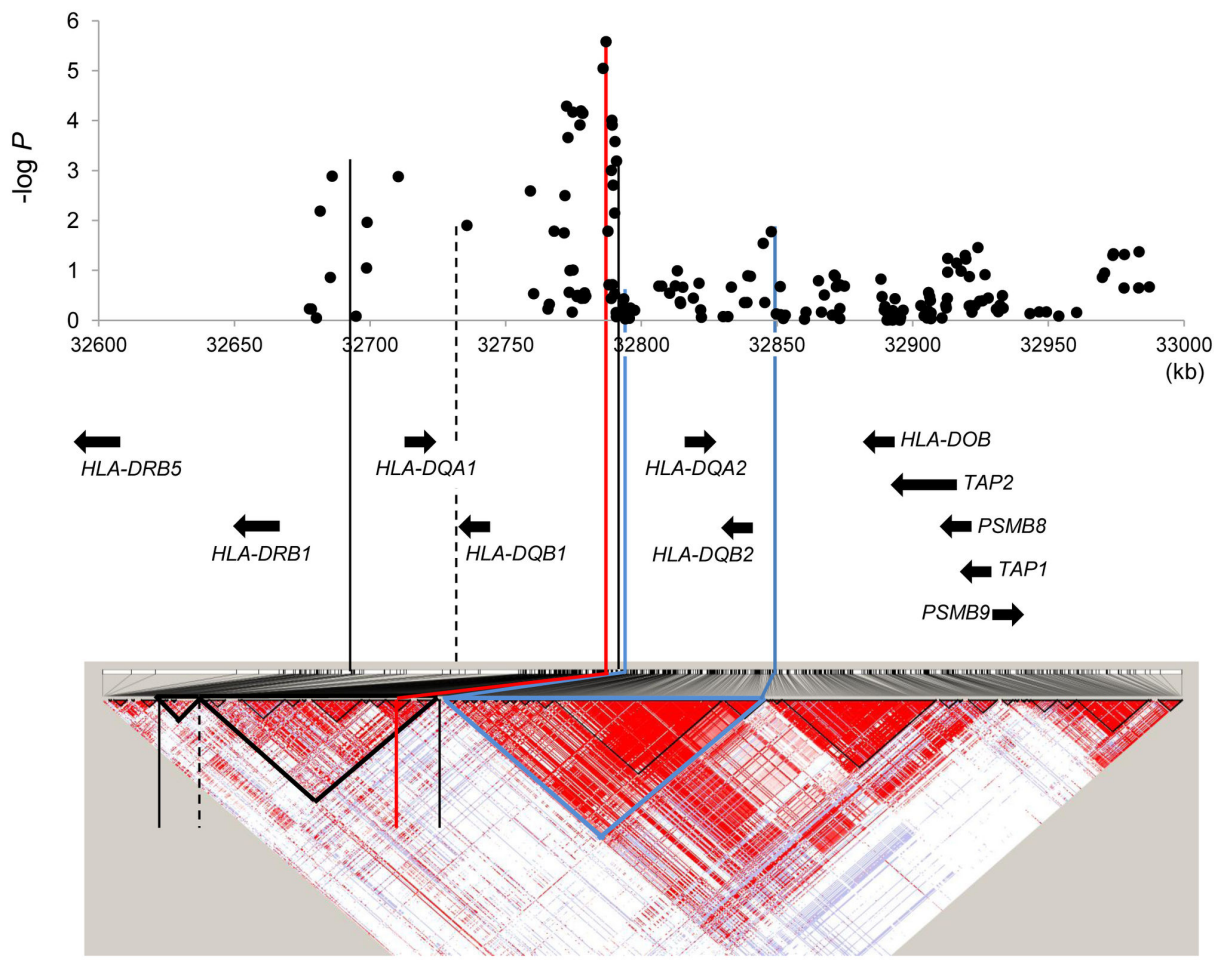
Linkage disequilibrium structure around the HLA-DQ region based on *D*’ using HapMap-JPT Data. P-value plot and genomic structure of the GWAS stage. Blue lines represent the LD block containing the *HLA-DQA2* and *HLA-DQB2* loci. Black lines represent two LD blocks containing the *HLA-DQA1* and *HLA-DQB1* loci, and the black dotted line represents the boundary between them. The red line represents the most strongly associated SNP, rs9275572, which is located within the *HLA-DQB1* locus. The LD maps were created using HaploView software.

Other than rs9275572, which is located within the HLA-DQ locus (Figure S2), several SNPs within the major histocompatibility complex (MHC) region showed suggestive associations but failed to be validated in a replication study. The linkage disequilibrium (LD) structure around the HLA-DQ region indicates that the landmark SNP is located in an LD block containing *HLA-DQB1* and *HLA-DQA1* (Figure 1 and Figure S3). Therefore, we next genotyped *HLA-DQA1* and *HLA-DQB1* alleles by direct sequencing of exon 2 using all 1,114 healthy controls of the first replication set and the same number of cases with HCV genotype 1 obtained from the same set and collected from the same geographical area. Over 95% of samples were successfully genotyped for each HLA-DQ, and common alleles with frequencies of more than 1 % were determined (7 and 11 alleles for *HLA-DQA1* and *HLA-DQB1*, respectively) (Table 2, Table S5A and S5B). Although no allele showed significant association (*P* < 0.05/7) among *HLA-DQA1* alleles, the HLA-DQB1*0303 allele showed significant association among *HLA-DQB1* alleles (*P* < 0.05/12, OR = 0.69). We also performed haplotype analysis using 1,025 cases and 1,038 controls that had been successfully genotyped for *HLA-DQA1*, *HLA-DQB1* and rs9275572 to investigate the effects of haplotype combinations; however, no haplotype showed stronger association than the single association of HLA-DQB1*0303 (Table S6). In our Japanese cohort, DQB1*0303 was the most frequent of the DQB1*03 alleles and showed the strongest effect against HCV persistence, but we also found that all DQB1*03 alleles tended to have a protective effect. Hence, we speculate that not only DQB1*0303, but all DQB1*03 alleles share protective residues. We searched for a variant that is common to all DQB1*03 alleles but that differs from non-DQB1*03 alleles and found that SNP rs1130380 is a variant specific to DQB1*03 (Table S5A and S5B). Then, we evaluated the single marker effect of several nucleotide variants, which are useful to distinguish among common DQB1 alleles using the same individuals with the haplotype analysis, and finally determined that rs1130380 (*P* = 6.08 × 10^−7^, OR = 0.72) had a stronger effect than the landmark SNP, rs9275572 (*P* = 2.07 × 10^−5^, OR = 0.76) (Table S7). After mutual adjustment between rs1130380 and rs9275572 using multiple logistic regression analysis, the association for both variants was remarkably attenuated (*P* = 2.38 × 10^−3^ and *P* = 0.0534, respectively), suggesting their mutual linkage (*D*’ = 0.99, *r* ^2^ = 0.28) (Figure S4). Therefore, these variants probably represent the same genetic signal and rs1130380 could be more compatible as a representative variant of the signal. This nucleotide polymorphism causes an amino acid substitution at position 55 on the HLA-DQB1 molecule. We examined the position of this amino acid substitution using on a three-dimensional structure of HLA-DQ molecule that was determined by X-ray diffraction method [21]. As shown in Figure S5, the amino acid at β55 forms part of a peptide-binding pocket in the HLA-DQ molecule, suggesting a critical role in antigen presentation. The change from Arg to Pro implies a physicochemcial change from basic to hydrophobic (Figure S6), but we have no relevant data with which to show a change of affinity to certain peptides at this time. Further functional analysis is therefore needed.

**Table 2:** Effect of common *HLA-DQA1* and *HLA-DQB1* haplotypes on chronic hepatitis C.

		**Case**			**Control**				
		**n**	**freq.(%)**		**n**	**freq.(%)**	**OR^a^**	**(95%CI)**	***P*^b^**
DQA1	*0101	304	14.3		320	14.4	0.99	(0.84-1.18)	9.53E-01
	*0102	302	14.2		263	11.8	1.24	(1.04-1.48)	1.84E-02
	*0103	445	21.0		423	19.0	1.13	(0.97-1.31)	1.08E-01
									
	*0301	830	39.1		924	41.5	0.90	(0.80-1.02)	1.02E-01
									
	*0401	65	3.1		58	2.6	1.18	(0.82-1.69)	3.66E-01
									
	*0501	138	6.5		177	8.0	0.80	(0.64-1.01)	6.44E-02
									
	*0601	30	1.4		49	2.2	0.64	(0.40-1.01)	5.15E-02
									
DQB1	*0301	181	8.4		207	9.6	0.86	(0.70-1.06)	1.69E-01
	*0302	220	10.2		245	11.4	0.89	(0.73-1.08)	2.24E-01
	*0303	272	12.6		372	17.3	0.69	(0.59-0.82)	2.05E-05
									
	*0401	305	14.2		282	13.1	1.10	(0.92-1.30)	3.01E-01
	*0402	114	5.3		84	3.9	1.38	(1.03-1.84)	2.86E-02
									
	*0501	147	6.8		168	7.8	0.87	(0.69-1.09)	2.22E-01
	*0502	59	2.7		61	2.8	0.97	(0.67-1.39)	8.57E-01
	*0503	116	5.4		78	3.6	1.52	(1.13-2.03)	5.14E-03
									
	*0601	444	20.6		409	19.0	1.11	(0.95-1.29)	1.76E-01
	*0602	155	7.2		128	5.9	1.23	(0.96-1.57)	9.52E-02
	*0604	119	5.5		98	4.5	1.23	(0.93-1.62)	1.42E-01
									

^a^Odds ratios of haplotype from two-by-two frequency table. ^b^ *P* value of chi-squared test.

Only alleles with frequencies of more than 1% were shown.

### Evaluation of *IFNL3-IFNL4* locus in GWAS

In spite of our failure to detect it in our GWAS screen, variants close to *IFNL3* have been reported to be associated with spontaneous clearance of HCV, so we tried to confirm the association using our GWAS data. Because genotype data for rs12979860 was not available in our platform, we checked the genotype data using another frequently reported SNP, rs8099917, which is highly linked with rs12979860 in the Japanese population (*r* ^2^ = 0.94) [22, 23]. As shown in Table S8, the association was confirmed in our GWAS set (*P* = 3.22 × 10^−3^, OR = 0.73) with the same allele conferring a protective effect.

### Analysis of *IFNL3-IFNL4* locus and HLA-DQ*03

We genotyped a *IFNL4* variant, ss469415590, and two representative variants close to *IFNL3*, rs12979860 and rs8099917, by Invader assay using the same individuals used in the detailed analysis of the HLA-DQ alleles above (1,114 cases and 1,114 controls). Each of these three polymorphisms were successfully genotyped in 1,074 cases and 1,073 controls. rs12979860 and ss469415590 were in almost complete LD, and ss469415590 showed the strongest association with HCV persistence under the additive model (*P* = 2.96 × 10^−3^, OR = 0.75) (Table 3). After adjusting for the effect of the HLA-DQ*03 allele using multiple logistic regression analysis, the significance level of ss469415590 increased slightly (*P* = 1.67 × 10^−3^, OR=0.74). We additionally analyzed the association under different genetic models other than additive. Among several genetic models, the *IFNL4* variant, ss469415590, showed the strongest association under the recessive model for the protective allele (the dominant model for the risk allele), whereas rs1130380 showed the strongest association under the allelic model (Table S9). We further evaluated the effect of the DQB1*03 allele stratified by ss469415590 genotype (Table 4). Interestingly, DQB1*03 showed a stronger effect in individuals with the ss469415590-TT/TT genotype, and this tendency became more pronounced for rs9275572, the landmark SNP in our GWAS (heterogeneity test *P* = 2.45 × 10^−3^).

**Table 3:** Case-control association analysis of 5 variants using 1,074 chronic hepatitis C cases and 1,073 healthy controls.

	**Allele**	**Case**				**Control**				**HWE-p^a^**			**Protective AF**				
SNP	[1/2]	11	12	22		11	12	22		Case	Control		Case	Control	OR^b^	(95%CI)	*P* ^c^
rs8099917	G/T	15	235	824		10	188	875		0.704	0.978		0.877	0.903	0.76	(0.63-0.92)	5.53E-03
rs12979860	T/C	16	244	814		11	194	868		0.637	0.965		0.872	0.899	0.76	(0.63-0.92)	4.08E-03
ss469415590	ΔG/TT	16	244	814		11	192	870		0.637	0.911		0.872	0.900	0.75	(0.62-0.91)	2.96E-03
																	
rs9275572	T/C	166	492	416		112	459	502		0.305	0.641		0.616	0.682	0.75	(0.66-0.85)	9.64E-06
rs1130380	C/G	120	433	521		172	478	423		0.039	0.060		0.313	0.383	0.73	(0.65-0.83)	3.38E-06
																	

AF; allele frequency, OR; odds ratio, CI; confidence interval.

^a^ *P* value of Hardy-Weinberg equilibrium test. ^b^Odds ratio of protective allele. ^c^ *P* value of Cochran-Armitage trend test.

**Table 4:** Effect of HLA-DQB1*03 and rs9275572 on the susceptibility of chronic hepatitis C stratified by genotype of ss469415590.

ss469415590	Allele	Case				Control				Protective AF					
	[1/2]	11	12	22		11	12	22		Case	Control	OR	(95%CI)^a^	*P* ^b^	*P*_het_^c^
TT/TT	*HLA-DQB1*	80	330	404		137	386	347		0.301	0.379	0.70	(0.61-0.81)	3.09E-06	3.36E-01
ΔG/TT or ΔG/ΔG	[*03 / non-*03]	40	103	117		35	92	76		0.352	0.399	0.82	(0.63-1.07)	1.61E-01	
TT/TT	rs9275572	134	389	291		89	367	414		0.596	0.687	0.67	(0.59-0.78)	6.31E-08	2.45E-03
ΔG/TT or ΔG/ΔG	[T / C]	32	103	125		23	92	88		0.679	0.660	1.09	(0.83-1.43)	5.56E-01	
															

AF; allele frequency, OR; odds ratio, CI; confidence interval.

^a^Odds ratios of protective allele from two-by-two allele frequency table. ^b^ *P* value of Cochran-Armitage trend test. ^c^Result of Breslow-Day test.

## Discussion

The MHC region containing the *HLA* locus is widely believed to play an important role in viral infection, and a recent GWAS revealed a strong association between the HLA-DP and *DQ* locus and hepatitis B virus (HBV) persistence [24, 25] as well as HBV-induced HCC [26] in East Asia. In our GWAS with two replication studies, we aimed to clarify the genetic factors involved in chronic HCV infection, and we successfully identified an association with the *HLA-DQB1* locus based on the landmark SNP rs9275572. An association between the MICA locus and the risk of developing HCV-induced HCC was previously reported in a Japanese GWAS [27], and this study identified rs9275572 as a second associated SNP. HCV-positive cases in the previous study, which are independent of ours, showed a similar minor allele frequency (MAF) of rs9275572 to our cases (0.407 in GWAS stage and 0.429 in replication stage), which strongly supports our results. Furthermore, according to the 1000 Genomes database, the MAF of this SNP is 0.348 in the Japanese population, which is consistent with the MAF of our controls.

On the other hand, while we observed no significant heterogeneity across the three studies, there were some differences in MAF among studies in both case and control populations. For both cases and controls, MAFs were lower in the first replication study than in the other two studies. Among the three studies, only the first replication set was collected from the same geographical area, therefore, geographical differences might affect the differences in MAF among studies, but this is only speculation. There are also differences in the gender and age distribution among studies (Table S10). Nonetheless, it is difficult to find a suitable explanation for the differences in MAF at this time.

In the current study, we failed to detect the previously most replicated *IFNL3-IFNL4* locus during GWAS screening even though the association indeed existed in our GWAS set (*P* = 3.22 × 10^−3^, OR = 0.73; Table S8). The odds ratio for rs8099917 is nearly the same as for rs9275572, but the P values were quite different, with rs9275572 ranked in the top 9 candidate SNPS, whereas the other is far below at number 1,640. This crucial difference may be explained by differences in allele frequencies. The minor allele frequency of rs9275572 is over three times higher than that of rs8099917, and the statistical power to detect each SNP in our GWAS was calculated to be only 65% and 8%, respectively, compared to the 80% recommended to detect an association of the expected effect size. We are concerned that there are several remaining undetected SNPs that might affect HCV persistence or clearance, in addition to the *IFNL3-IFNL4* locus. To overcome this problem, it will be necessary to use a larger sample size, as well as more appropriate control samples. In fact, the major limitation of our study involves the selection of controls. Undoubtedly, the most appropriate control set would include patients who have been exposed to HCV (anti-HCV positive) but who spontaneously cleared the infection (HCV-RNA negative). Although the risk of exposure to HCV in the Japanese general population is very low, making it difficult to locate suitable control subjects, we admit concede that our choice of study design has reduced the statistical power of the study and might have undermined the conclusions.

HLA-DQs belong to the HLA class II molecules that form α-β heterodimers on the cell surface of antigen-presenting cells, such as macrophages, dendritic cells and B lymphocytes. HLA-DQ genes play a central role in immune-mediated diseases by presenting peptides derived from extracellular proteins to CD4-positive T lymphocytes. Both *HLA-DQB1* and *HLA-DQA1* are highly polymorphic, especially in exon 2, which encodes antigen-binding sites that confer binding specificity. We speculated that the association of the landmark SNP with chronic hepatitis C might reﬂect variation in HLA-DQ antigen-binding sites and that somewhat different effects of rs9275572 between HCV genotypes might reflect differences in binding affinities between the HLA-DQ pocket and HCV genotype-specific epitopes (Table S4B). Finally, we found that a single amino acid polymorphism (R55P) corresponding to DQB1*03, a very common allele worldwide [28], has the strongest protective effect against HCV persistence. Among DQB1*03 alleles, DQB1*0301 has the highest frequency various populations worldwide and has been reported in association with viral clearance in several European and American studies [7, 13]. A suggestive paper by Cangussu et al. found an association between protection against HCV persistence and not only DQB1*0301 but also DQB1*0302 and DQB1*0303 in a white Brazilian cohort [29]. They suggested that HLA-DQ beta molecules responsible for protection are present not only in molecules encoded by DQB1*0301 but also by DQB1*0302 and DQB1*0303, and the common polymorphic residues shared by DQB1*03 alleles are responsible for the selection of particular HCV epitopes. In the current study, we identified that not only the allele with the strongest effect, DQB1*0303, but all DQB1*03 alleles share protective residues at position 55 in at least the Japanese and white Brazilian populations. Although the human MHC region encompasses a complex and extended LD structure, and LD among SNPs in the HLA-class II locus is known to vary among ethnic populations, various immunological functions of MHC molecules have gradually become clear. Several issues had to be overcome in order improve our understanding of whether or not the HLA-DQB1 molecule has common functions across multiple populations.

In this study, we also made an intriguing observation that HLA-DQB1*03 showed a stronger effect in individuals with the ss469415590-TT/TT genotype (Table 4), suggesting that the effect of HLA-DQB1*03 on HCV persistence might be affected by the genotype of the *IFNL4* polymorphism. The fact that in the Japanese population the frequency of the ss469415590-TT/TT genotype is nearly equal to that of the rs12979860-CC genotype (JPT 78.7%, CEU 57.6%, AFR 15.4% based on the 1000 Genomes database), with a higher frequency than most other populations, might partially explain the differences among the top loci in European versus Japanese GWAS results. Finally, we determined the relationship between HLA-DQB1*03 (rs1130380) and *IFNL4* variants (ss469415590) in cases and controls (Figure S7A and S7B). In cases, the proportion of individuals with the protective ss469415590-TT/TT genotype significantly increased while the proportion of protective DQB1*03 alleles decreased, but this tendency was not observed in controls. During any phase of HCV infection, these two polymorphisms might affect each other in complementary or exclusive fashion.

A recent GWAS reported that variants close to *IFNL3* and DQB1*0301 are independently associated with spontaneous resolution of HCV infection [13]. On the other hand, our study newly suggests that a single amino acid polymorphism, common throughout the world, at position 55 on the HLA-DQB1 molecule has the strongest association with chronic HCV infection and may not be independent of the *IFNL4* polymorphism.

In summary, we conducted a GWAS followed by two independent replication studies and detected genetic variants in the HLA-DQ genes strongly associated with chronic hepatitis C in the Japanese population. Our findings suggest that variation in antigen-binding sites involved in antigen presentation on HLA-DQ molecules might play an important role in HCV persistence or clearance. Further research is required to determine the distinct roles of HLA-DQB1 and IFNL4 in chronic HCV infection.

## Materials and Methods

### Study Population

Characteristics of each case-control group are shown in Table S10. Case samples used in this study were obtained between 2002 and 2012 from Toranomon Hospital Department of Hepatology, Sapporo Kosei General Hospital and Hiroshima University-affiliated hospitals. We used 481 samples from Toranomon Hospital as cases for the GWAS and the remaining 1,004 samples, along with 375 samples from Sapporo Kosei General Hospital, as cases for the second replication study. We used all 4,358 samples from Hiroshima University-affiliated hospitals as cases for the first replication study. All cases had abnormal levels of serum alanine aminotransferase for more than 6 months and were positive for both anti-HCV and serum HCV RNA. All patients were negative for hepatitis B surface antigen (HBsAg), had no evidence of other liver diseases, and had not received immunosuppressive therapy before enrollment. Control samples used for the GWAS and second replication studies were obtained from BioBank Japan at the Institute of Medical Science, University of Tokyo [30]. The control groups included 2,057 individuals for the GWAS and 25,817 independent samples for the second replication study and consisted of GWAS samples for unrelated diseases in the BioBank Japan project. We excluded samples that were positive for HBsAg or anti-HCV or that were registered as positive for any liver disease. We also obtained DNA from 906 Japanese control subjects from volunteers without liver disease in the Osaka-Midosuji Rotary Club, Osaka, Japan. In addition, we obtained 1,114 samples as healthy controls for the second replication study from volunteers without any liver diseases at Hiroshima University, Hiroshima, Japan.

### Ethics statement

All subjects received a detailed explanation, and all signed a written informed consent form. The study protocol conforms to the ethical guidelines of the 1975 Declaration of Helsinki and was approved a priori by the ethical committees at University of Tokyo, Hiroshima University, Toranomon Hospital and Sapporo Kosei General Hospital and by the Ethical Committee at the SNP Research Center, the Institute of Physical and Chemical Research (RIKEN), Yokohama.

### SNP Genotyping and Quality Control

Genomic DNA was extracted from peripheral blood leukocytes using a standard method. For the GWAS stage, we genotyped 496 cases using the Illumina HumanHap610-Quad BeadChip (San Diego, CA, USA). We excluded 2 samples with call rates < 0.98. 13 other samples with apparent kinship or sample duplication were excluded from the analysis based on PI_HAT values (> 0.4). We assessed population stratification using the smartpca program in the EIGENSOFT package using SNPs informative for the Japanese population according to a previously described method [31]. Analysis was performed based on the GWAS data and the Japanese (JPT), Han-Chinese (CHB), European (CEU), and African (YRI) individuals from the International HapMap Project. Principal component analysis identified no outliers from the JPT/CHB clusters. 458,207 autosomal SNPs passed the quality control filters (call rate ≥ 0.99 in both cases and controls, a MAF > 0 and a Hardy-Weinberg equilibrium *P* value ≥ 1.0 × 10^−6^ in controls). We used multiplex-PCR-based Invader assays (Third Wave Technologies, Madison, WI, USA) for the replication studies [32]. Samples for both cases and controls were distributed randomly on genotyping plates in both phases of the study, and all persons performing genotyping and interpretation of results were blind to case / control status.

### *HLA-DQA1* and *HLA-DQB1* Genotyping

We analyzed HLA-DQ genotypes using 1,114 cases and 1,114 controls in the ﬁrst replication set. Exon 2 of the *HLA-DQA1* and *HLA-DQB1* genes were ampliﬁed and directly sequenced following the protocol of the International Histocompatibility Workshop Group [33]. *HLA-DQA1* and DQB1 alleles were determined based on the alignment database of dbMHC (http://www.ncbi.nlm.nih.gov/gv/mhc/align.cgi?cmd=aligndisplay&locus_name=HLA-DQB1&banner=1#).

### Protein Analysis

To represent the position of amino acid substitution caused by the SNP, we edited the image of a three-dimensional structure of HLA-DQ molecule that was previously determined by X-ray diffraction method [21] using Protein Data Bank Japan (PDBj) Viewer (http://www.pdbj.org/index_j.html). The amino acid numbering excludes the 32 amino acid signal peptide.

### Statistical Analysis

Genotype-based associations were tested using the Cochran-Armitage trend test. ORs and CIs were calculated from a two-by-two allele frequency table. Combined analysis was performed following the Mantel-Haenszel method. Heterogeneity among studies was examined using the Breslow-Day test. We used HaploView software to analyze the association of haplotypes and LD values between *HLA-DQs* and SNPs [34]. Chi-squared or Fisher exact tests were used to analyze categorical data, as appropriate. For general statistical analysis, we used the R statistical environment version 2.12.0 (http://www.cran.r-project.org/) or PLINK 1.06 (http://pngu.mgh.harvard.edu/~purcell/plink/) [35]. Power analysis was performed using Power for Genetic Association Analyses software [36]. SNP allele frequencies were collected from the 1000 Genomes database (http://browser.1000genomes.org/).

## Supporting Information

Figure S1 **Results of the GWAS.** (A) Quantile-quantile plot for the Cochran-Armitage trend tests for the GWAS phase. The horizontal axis represents *P* values expected under a null distribution, and the vertical axis shows the observed *P* values. Under the null hypothesis of no association at any locus, the points would be expected to fall along the line (y=x). (B) Manhattan plot showing the -log_10_ *P* value of each SNP calculated using the 1-d.f. Cochran-Armitage trend test. The red line shows the Bonferroni cutoff for genome-wide significance (*P* = 1.09×10^−7^) given the number of SNPs analyzed in this study (0.05/458207).
(TIF)

Figure S2 **Case-control association results of the MHC region.** P-value plot and genomic structure of the GWAS stage within the extended MHC region of chromosome 6. The black dotted line represents the SNP with the strongest association, rs9275572, which is located within the *HLA-DQ* locus.
(TIF)

Figure S3 **Linkage disequilibrium structure around the *HLA-DQ* region based on *D’* and *r*^*2*^ using GWAS data.** The red line represents the most strongly associated SNP, rs9275572, which is located within the *HLA-DQB1* locus. The LD maps were created using HaploView software.
(TIF)

Figure S4 **Linkage disequilibrium structure between rs1130380 and rs9275572 based on *D’* and *r*^*2*^ using data from 2,063 individuals (1,025 cases and 1,038 controls).** The LD maps were created using HaploView software.
(TIF)

Figure S5 **The position of the amino acid at β55 on the HLA-DQ molecule.** An edited representation of the three-dimensional structure of HLA-DQ molecule that was previously determined by X-ray diffraction method [21] is shown. Protein Data Bank Japan (PDBj) Viewer (http://www.pdbj.org/index_j.html) was used for editing. The alpha and beta chains are represented by green and yellow, respectively. The amino acid at β55 is indicated as red. Amino acid numbering excludes the 32 amino acid signal peptide.
(TIF)

Figure S6 **Chromatograms of different alleles of rs1130380 and their predicted effects on protein translation.** Two different PCR amplifications were performed for genotyping of *HLA-DQB1* alleles: amplicon 1 for the *02/03/04* alleles and amplicon 2 for the *05/06* alleles [33]. The G to C nucleotide change of codon 55 leads to amino acid substitution from Arg to Pro. The properties of Arg and Pro are basic and hydrophobic, respectively. We also observed the G to T nucleotide change, but this variant was not considered for further analysis because of its low frequency (< 1%).
(TIF)

Figure S7 Relationship between HLA-DQB1*03 (rs1130380) and *IFNL4* variant (ss469415590) in chronic HCV patients (A) and healthy controls (B). Proportion of individuals with ss469415590 TT/TT in cases and controls according to HLA-DQB1*03 status. *P* values were calculated using the chi-squared test.
(TIF)

Table S1 **Results of GWAS.**
(PDF)

Table S2 **Results of 1st replication study.**
(PDF)

Table S3 **Multiple logistic regression analysis for the risk of chronic hepatitis C using 1st replication samples (4,347 cases and 1,097 controls).**
(PDF)

Table S4 **(A) Effect of rs9275572 on the susceptibility of chronic hepatitis C stratified by gender and age using 1st replication samples.** (B) Effect of rs9275572 on the susceptibility of chronic hepatitis C stratified by HCV genotype using 1st replication samples.
(PDF)

Table S5 **(A) Useful variants for genotyping common *HLA-DQA1* haplotypes in the Japanese population.** (B) Useful variants for genotyping common *HLA-DQB1* haplotypes in the Japanese population.
(PDF)

Table S6 **Haplotype analysis was performed using the landmark SNP (rs9275572) and 12 variants shown in Table S5A and S5B by HaploView software.**
(PDF)

Table S7 **Single marker effect on chronic hepatitis C.**
(PDF)

Table S8 **Results of GWAS of two SNPs.**
(PDF)

Table S9 **Case-control association analysis of 5 variants under different genetic models.**
(PDF)

Table S10 **Basic characteristics of study population.**
(PDF)

## References

[1] Chayama K, Hayes CN, Ohishi W, Kawakami Y (2013) Treatment of chronic hepatitis C virus infection in Japan: update on therapy and guidelines. J Gastroenterol 48: 1-12. doi:10.1007/s00535-012-0714-9. PubMed: 23188091. 23188091 PMC3698425

[2] Lavanchy D (2009) The global burden of hepatitis C. Liver Int 29 Suppl 1 : 74-81. doi:10.1111/j.1478-3231.2008.01702.x. PubMed: 19207969. 19207969

[3] Barrera JM, Bruguera M, Ercilla MG, Gil C, Celis R et al. (1995) Persistent hepatitis C viremia after acute self-limiting posttransfusion hepatitis C. Hepatology 21: 639-644. doi:10.1002/hep.1840210306. PubMed: 7533121. 7533121

[4] Welzel TM, Morgan TR, Bonkovsky HL, Naishadham D, Pfeiffer RM et al. (2009) Variants in interferon-alpha pathway genes and response to pegylated interferon-Alpha2a plus ribavirin for treatment of chronic hepatitis C virus infection in the hepatitis C antiviral long-term treatment against cirrhosis trial. Hepatology 49: 1847-1858. doi:10.1002/hep.22877. PubMed: 19434718. 19434718 PMC2692559

[5] Shepard CW, Finelli L, Alter MJ (2005) Global epidemiology of hepatitis C virus infection. Lancet Infect Dis 5: 558-567. doi:10.1016/S1473-3099(05)70216-4. PubMed: 16122679. 16122679

[6] Prieto M, Olaso V, Verdú C, Córdoba J, Gisbert C et al. (1995) Does the healthy hepatitis C virus carrier state really exist? An analysis using polymerase chain reaction. Hepatology 22: 413-417. doi:10.1016/0270-9139(95)95374-4. PubMed: 7635408. 7635408

[7] Singh R, Kaul R, Kaul A, Khan K (2007) A comparative review of HLA associations with hepatitis B and C viral infections across global populations. World J Gastroenterol 13: 1770-1787. PubMed: 17465466. 17465466 10.3748/wjg.v13.i12.1770 PMC4149952

[8] Kuzushita N, Hayashi N, Moribe T, Katayama K, Kanto T et al. (1998) Influence of HLA haplotypes on the clinical courses of individuals infected with hepatitis C virus. Hepatology 27: 240-244. doi:10.1002/hep.510270136. PubMed: 9425943. 9425943

[9] Yoshizawa K, Ota M, Saito S, Maruyama A, Yamaura T et al. (2003) Long-term follow-up of hepatitis C virus infection: HLA class II loci influences the natural history of the disease. Tissue Antigens 61: 159-165. doi:10.1034/j.1399-0039.2003.00015.x. PubMed: 12694584. 12694584

[10] Khakoo SI, Thio CL, Martin MP, Brooks CR, Gao X et al. (2004) HLA and NK cell inhibitory receptor genes in resolving hepatitis C virus infection. Science 305: 872-874. doi:10.1126/science.1097670. PubMed: 15297676. 15297676

[11] Thomas DL, Thio CL, Martin MP, Qi Y, Ge D et al. (2009) Genetic variation in IL28B and spontaneous clearance of hepatitis C virus. Nature 461: 798-801. doi:10.1038/nature08463. PubMed: 19759533. 19759533 PMC3172006

[12] Rauch A, Kutalik Z, Descombes P, Cai T, Di Iulio J, et al. (2010) Genetic variation in IL28B is associated with chronic hepatitis C and treatment failure: a genome-wide association study. Gastroenterology 138: 1338-1345.e7 10.1053/j.gastro.2009.12.056 20060832

[13] Duggal P, Thio CL, Wojcik GL, Goedert JJ, Mangia A et al. (2013) Genome-wide association study of spontaneous resolution of hepatitis C virus infection: data from multiple cohorts. Ann Intern Med 158: 235-245. doi:10.7326/0003-4819-158-4-201302190-00003. PubMed: 23420232. 23420232 PMC3638215

[14] Prokunina-Olsson L, Muchmore B, Tang W, Pfeiffer RM, Park H et al. (2013) A variant upstream of IFNL3 (IL28B) creating a new interferon gene IFNL4 is associated with impaired clearance of hepatitis C virus. Nat Genet 45: 164-171. doi:10.1038/ng.2521. PubMed: 23291588. 23291588 PMC3793390

[15] Tanaka J, Kumagai J, Katayama K, Komiya Y, Mizui M et al. (2004) Sex- and age-specific carriers of hepatitis B and C viruses in Japan estimated by the prevalence in the Mart,485,648 first-time blood donors during 1995-2000. Intervirology 47: 32-40. doi:10.1159/000076640. PubMed: 15044834. 15044834

[16] Bakr I, Rekacewicz C, El Hosseiny M, Ismail S, El Daly M et al. (2006) Higher clearance of hepatitis C virus infection in females compared with males. Gut 55: 1183-1187. PubMed: 16434426. 16434426 10.1136/gut.2005.078147 PMC1856273

[17] Kasahara A, Hayashi N, Mochizuki K, Takayanagi M, Yoshioka K et al. (1998) Risk factors for hepatocellular carcinoma and its incidence after interferon treatment in patients with chronic hepatitis C. Osaka Liver Disease Study Group. Hepatology 27: 1394-1402. doi:10.1002/hep.510270529. PubMed: 9581697. 9581697

[18] Haydon GH, Jarvis LM, Simmonds P, Harrison DJ, Garden OJ et al. (1997) Association between chronic hepatitis C infection and hepatocellular carcinoma in a Scottish population. Gut 40: 128-132. PubMed: 9155590. 9155590 10.1136/gut.40.1.128 PMC1027022

[19] Kawaoka T, Hayes CN, Ohishi W, Ochi H, Maekawa T et al. (2011) Predictive value of the IL28B polymorphism on the effect of interferon therapy in chronic hepatitis C patients with genotypes 2a and 2b. J Hepatol 54: 408-414. doi:10.1016/j.jhep.2010.07.032. PubMed: 21112660. 21112660

[20] Hiraga N, Imamura M, Hatakeyama T, Kitamura S, Mitsui F et al. (2009) Absence of viral interference and different susceptibility to interferon between hepatitis B virus and hepatitis C virus in human hepatocyte chimeric mice. J Hepatol 51: 1046-1054. doi:10.1016/j.jhep.2009.09.002. PubMed: 19853955. 19853955

[21] Siebold C, Hansen BE, Wyer JR, Harlos K, Esnouf RE et al. (2004) Crystal structure of HLA-DQ0602 that protects against type 1 diabetes and confers strong susceptibility to narcolepsy. Proc Natl Acad Sci U S A 101: 1999-2004. doi:10.1073/pnas.0308458100. PubMed: 14769912. 14769912 PMC357041

[22] Ochi H, Maekawa T, Abe H, Hayashida Y, Nakano R et al. (2011) IL-28B predicts response to chronic hepatitis C therapy--fine-mapping and replication study in Asian populations. J Gen Virol 92: 1071-1081. doi:10.1099/vir.0.029124-0. PubMed: 21228123. 21228123

[23] Chayama K, Hayes CN, Imamura M (2012) Impact of interleukin-28B genotype on in vitro and in vivo systems of hepatitis C virus replication. Hepatol Res 42: 841-853. doi:10.1111/j.1872-034X.2012.01002.x. PubMed: 22524382. 22524382

[24] Mbarek H, Ochi H, Urabe Y, Kumar V, Kubo M et al. (2011) A genome-wide association study of chronic hepatitis B identified novel risk locus in a Japanese population. Hum Mol Genet 20: 3884-3892. doi:10.1093/hmg/ddr301. PubMed: 21750111. 21750111

[25] Kamatani Y, Wattanapokayakit S, Ochi H, Kawaguchi T, Takahashi A et al. (2009) A genome-wide association study identifies variants in the HLA-DP locus associated with chronic hepatitis B in Asians. Nat Genet 41: 591-595. doi:10.1038/ng.348. PubMed: 19349983. 19349983

[26] Jiang DK, Sun J, Cao G, Liu Y, Lin D et al. (2013) Genetic variants in STAT4 and HLA-DQ genes confer risk of hepatitis B virus-related hepatocellular carcinoma. Nat Genet 45: 72-75. doi:10.1038/ng.2483. PubMed: 23242368. 23242368 PMC4105840

[27] Kumar V, Kato N, Urabe Y, Takahashi A, Muroyama R et al. (2011) Genome-wide association study identifies a susceptibility locus for HCV-induced hepatocellular carcinoma. Nat Genet 43: 455-458. doi:10.1038/ng.809. PubMed: 21499248. 21499248

[28] Solberg OD, Mack SJ, Lancaster AK, Single RM, Tsai Y et al. (2008) Balancing selection and heterogeneity across the classical human leukocyte antigen loci: a meta-analytic review of 497 population studies. Hum Immunol 69: 443-464. doi:10.1016/j.humimm.2008.05.001. PubMed: 18638659. 18638659 PMC2632948

[29] Cangussu LO, Teixeira R, Campos EF, Rampim GF, Mingoti SA et al. (2011) HLA class II alleles and chronic hepatitis C virus infection. Scand J Immunol 74: 282-287. doi:10.1111/j.1365-3083.2011.02568.x. PubMed: 21535077. 21535077

[30] Nakamura Y (2007) The BioBank Japan Project. Clin Adv Hematol Oncol 5: 696-697. PubMed: 17982410. 17982410

[31] Yamaguchi-Kabata Y, Nakazono K, Takahashi A, Saito S, Hosono N et al. (2008) Japanese population structure, based on SNP genotypes from 7003 individuals compared to other ethnic groups: effects on population-based association studies. Am J Hum Genet 83: 445-456. doi:10.1016/j.ajhg.2008.08.019. PubMed: 18817904. 18817904 PMC2561928

[32] Ohnishi Y, Tanaka T, Ozaki K, Yamada R, Suzuki H et al. (2001) A high-throughput SNP typing system for genome-wide association studies. J Hum Genet 46: 471-477. doi:10.1007/s100380170047. PubMed: 11501945. 11501945

[33] van der Zwan A, Grifﬁth B, Rozemuller E, Williams T, Tilanus MGJ, Tilanus MGJ, ed. (2002) IHWG Technical Manual Genomic Analysis of the Human MHC: DNA-Based Typing for HLA Alleles and Linked Polymorphisms. Seattle: International Histocompatibility Working Group.

[34] Barrett JC, Fry B, Maller J, Daly MJ (2005) Haploview: analysis and visualization of LD and haplotype maps. Bioinformatics 21: 263-265. doi:10.1093/bioinformatics/bth457. PubMed: 15297300. 15297300

[35] Purcell S, Neale B, Todd-Brown K, Thomas L, Ferreira MA et al. (2007) PLINK: a tool set for whole-genome association and population-based linkage analyses. Am J Hum Genet 81: 559-575. doi:10.1086/519795. PubMed: 17701901. 17701901 PMC1950838

[36] Menashe I, Rosenberg PS, Chen BE (2008) PGA: power calculator for case-control genetic association analyses. BMC Genet 9: 36. doi:10.1186/1471-2350-9-36. PubMed: 18477402. 18477402 PMC2387159

