# Supplementary figures and images for: HLA-DQB1*03 Confers Susceptibility to Chronic Hepatitis C in Japanese: A Genome-Wide Association Study
Source: PLoS One. 2013 Dec 20;8(12):e84226. doi: 10.1371/journal.pone.0084226 (PMC3871580; doi:10.1371/journal.pone.0084226)

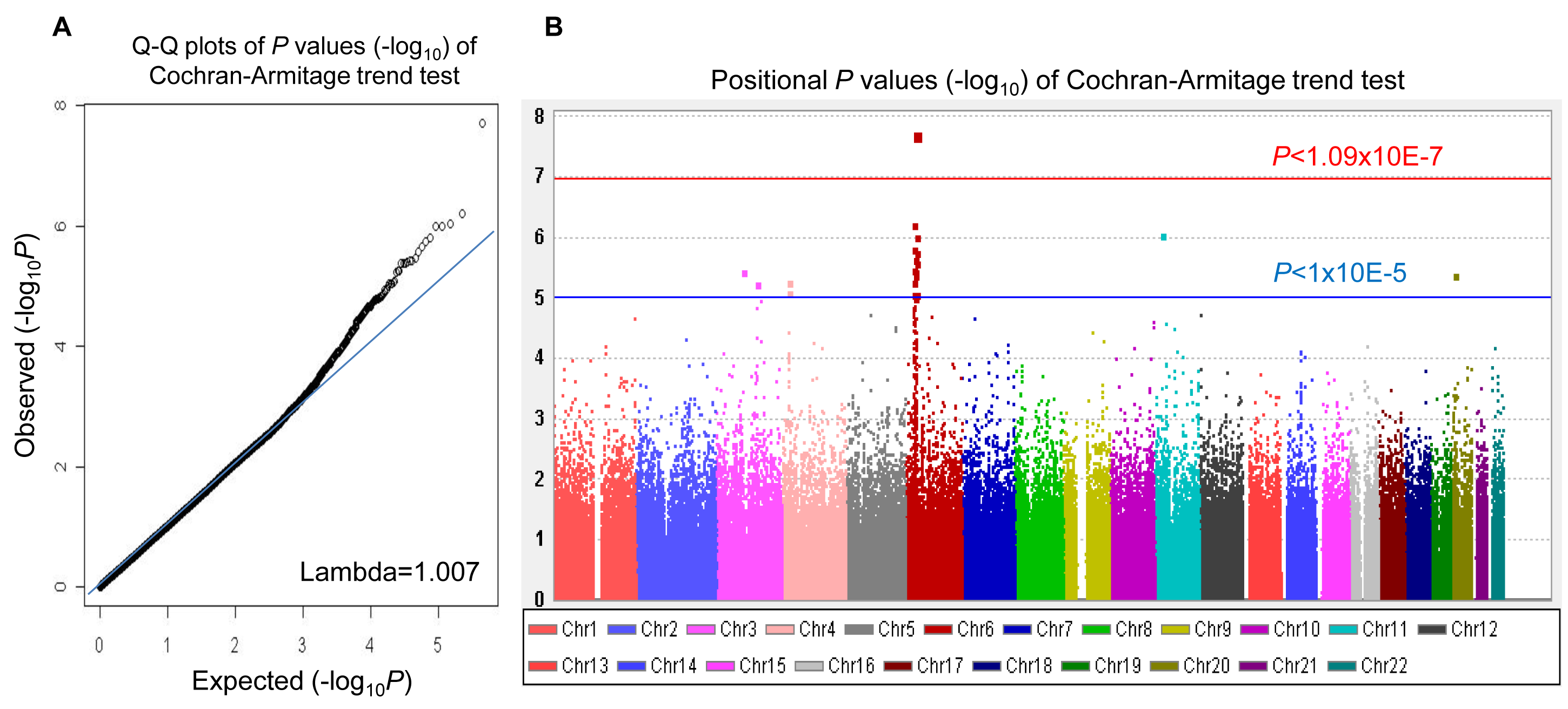

Supplement: Figure S1 — Results of the GWAS. (A) Quantile-quantile plot for the Cochran-Armitage trend tests for the GWAS phase. The horizontal axis represents P values expected under a null distribution, and the vertical axis shows the observed P values. Under the null hypothesis of no association at any locus, the points would be expected to fall along the line (y=x). (B) Manhattan plot showing the -log10 P value of each SNP calculated using the 1-d.f. Cochran-Armitage trend test. The red line shows the Bonferroni cutoff for genome-wide significance (P = 1.09×10^−7^) given the number of SNPs analyzed in this study (0.05/458207). (TIF) [file pone.0084226.s001.tif]

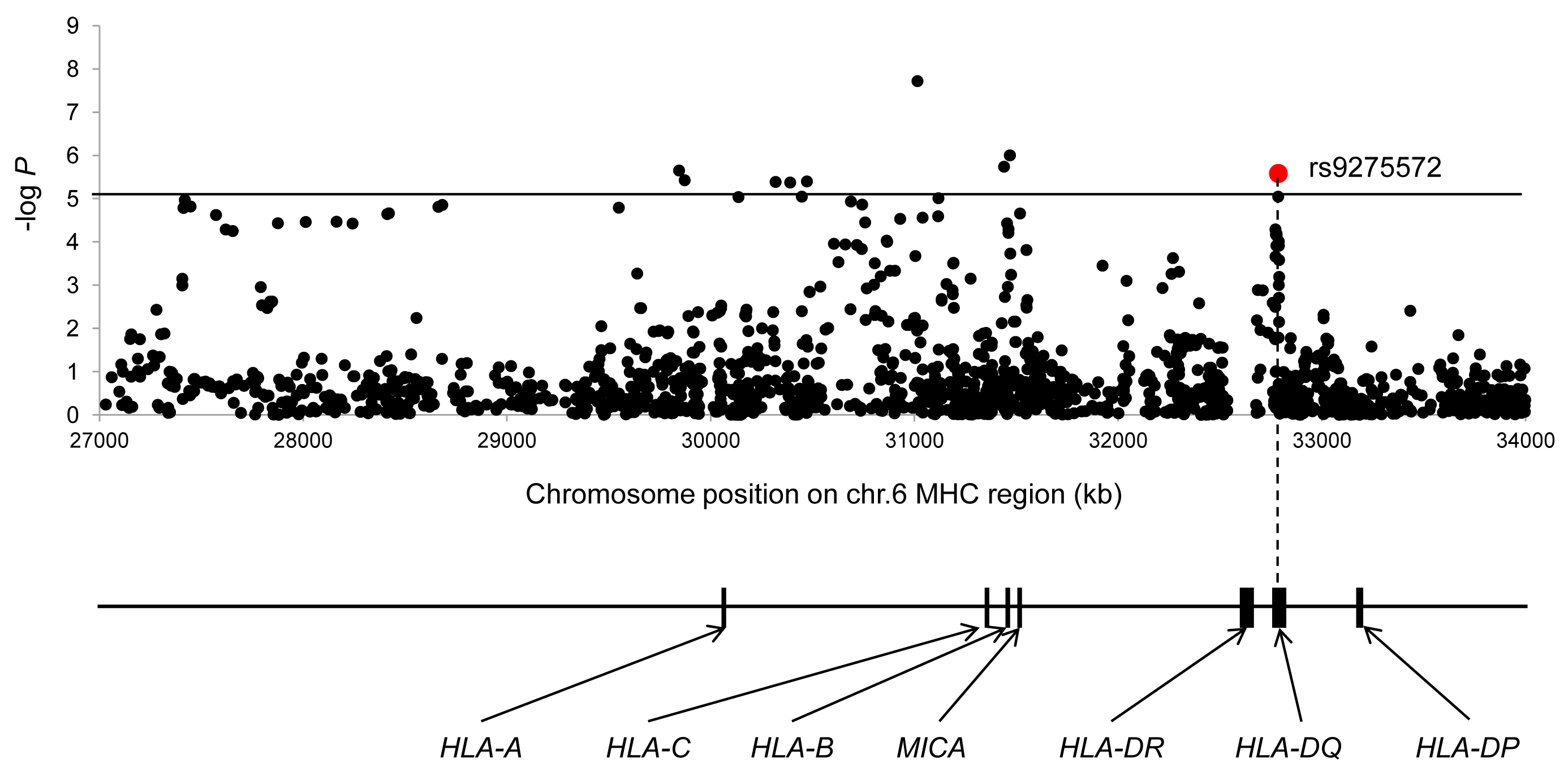

Supplement: Figure S2 — Case-control association results of the MHC region. P-value plot and genomic structure of the GWAS stage within the extended MHC region of chromosome 6. The black dotted line represents the SNP with the strongest association, rs9275572, which is located within the HLA-DQ locus. (TIF) [file pone.0084226.s002.tif]

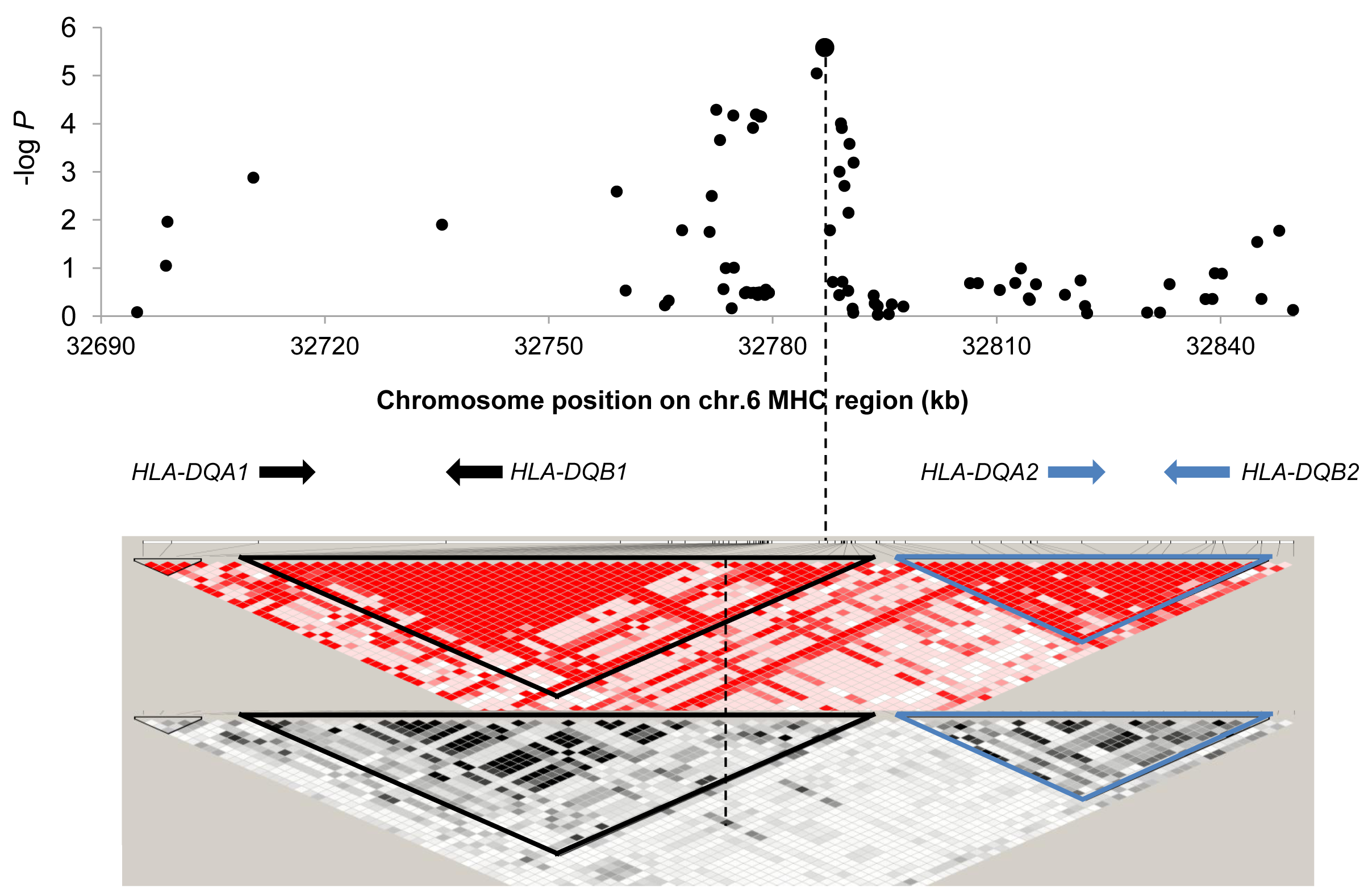

Supplement: Figure S3 — Linkage disequilibrium structure around the HLA-DQ region based on D’ and r2 using GWAS data. The red line represents the most strongly associated SNP, rs9275572, which is located within the HLA-DQB1 locus. The LD maps were created using HaploView software. (TIF) [file pone.0084226.s003.tif]

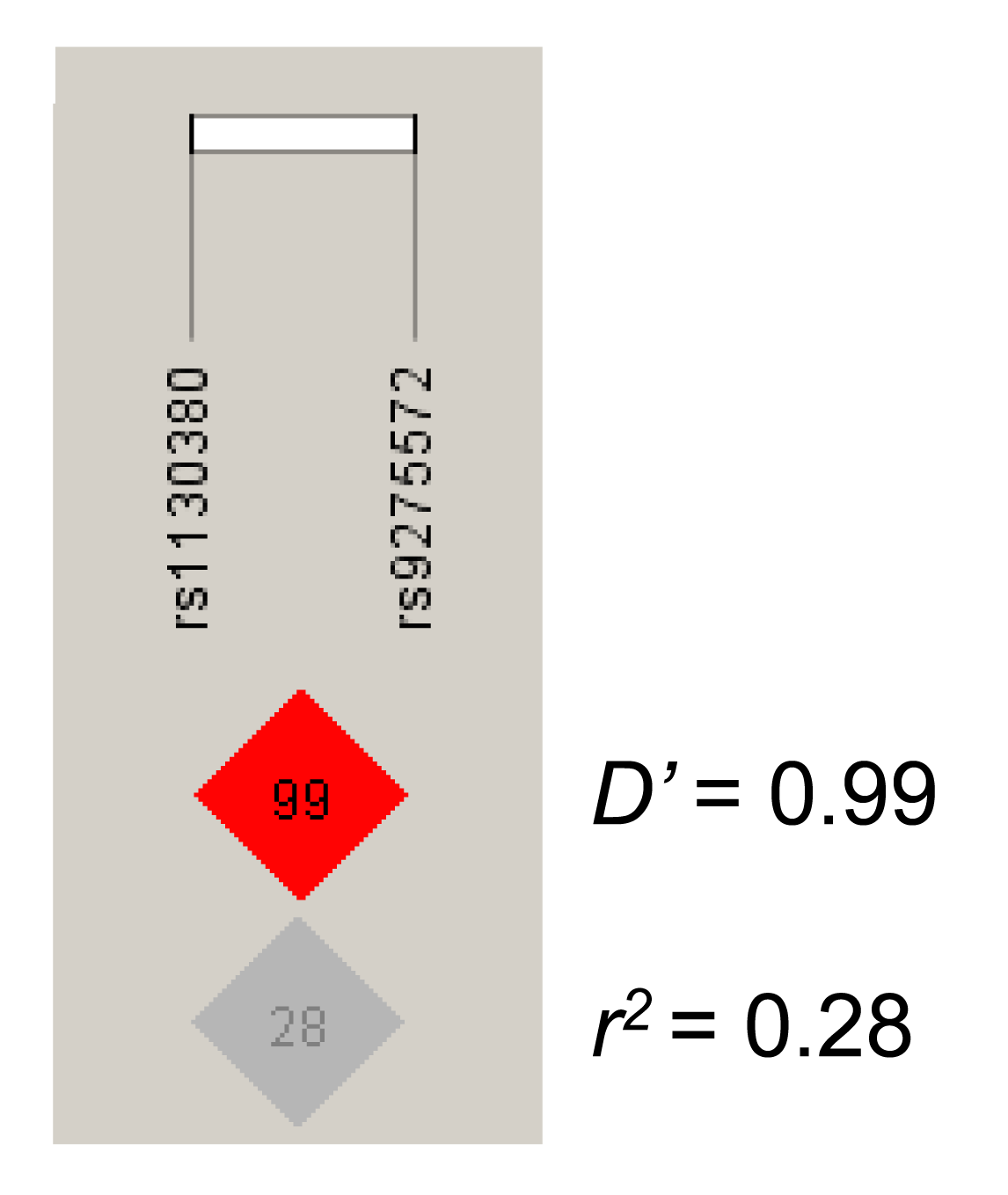

Supplement: Figure S4 — Linkage disequilibrium structure between rs1130380 and rs9275572 based on D’ and r2 using data from 2,063 individuals (1,025 cases and 1,038 controls). The LD maps were created using HaploView software. (TIF) [file pone.0084226.s004.tif]

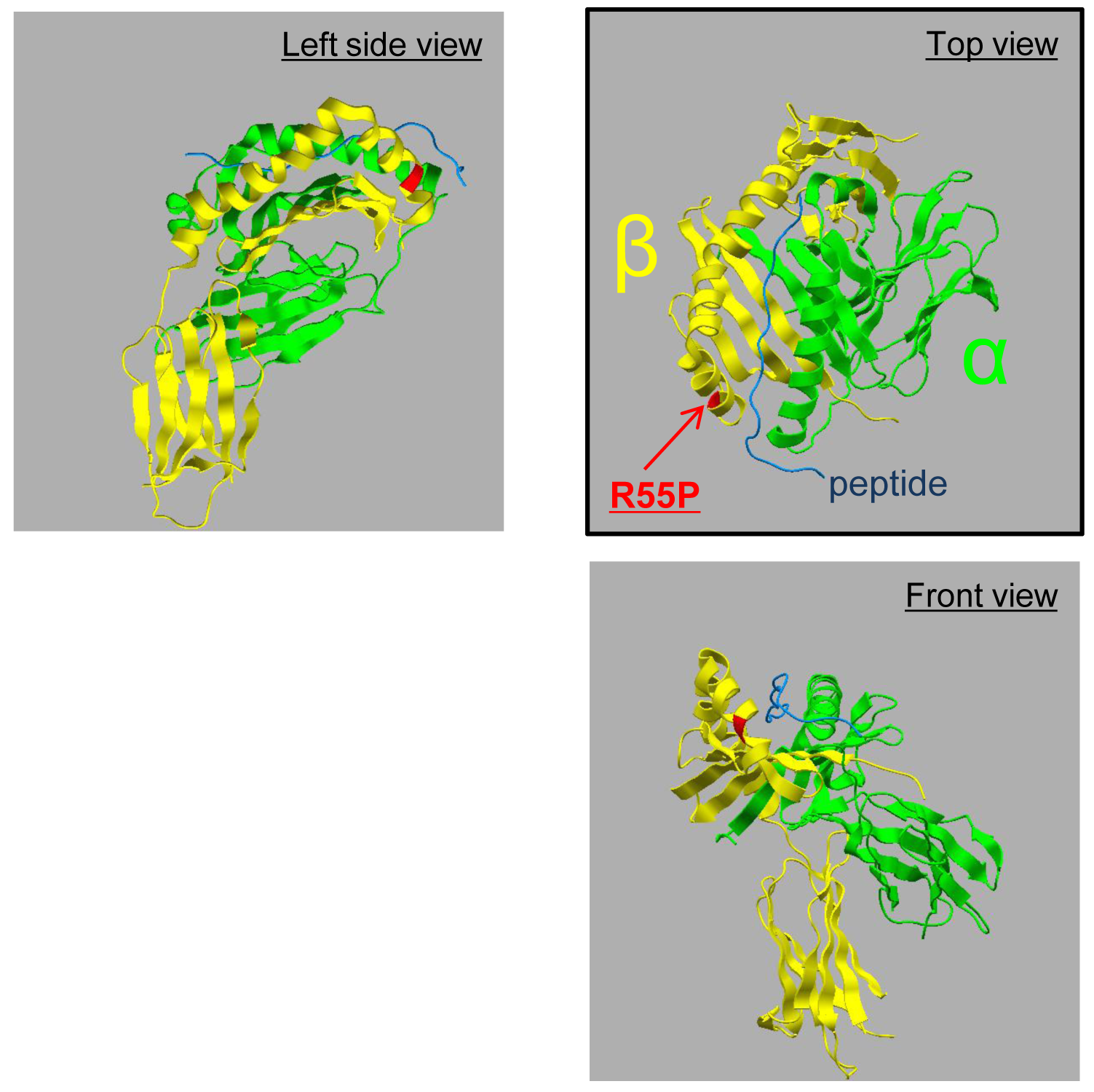

Supplement: Figure S5 — The position of the amino acid at β55 on the HLA-DQ molecule. An edited representation of the three-dimensional structure of HLA-DQ molecule that was previously determined by X-ray diffraction method [21] is shown. Protein Data Bank Japan (PDBj) Viewer (http://www.pdbj.org/index_j.html) was used for editing. The alpha and beta chains are represented by green and yellow, respectively. The amino acid at β55 is indicated as red. Amino acid numbering excludes the 32 amino acid signal peptide. (TIF) [file pone.0084226.s005.tif]

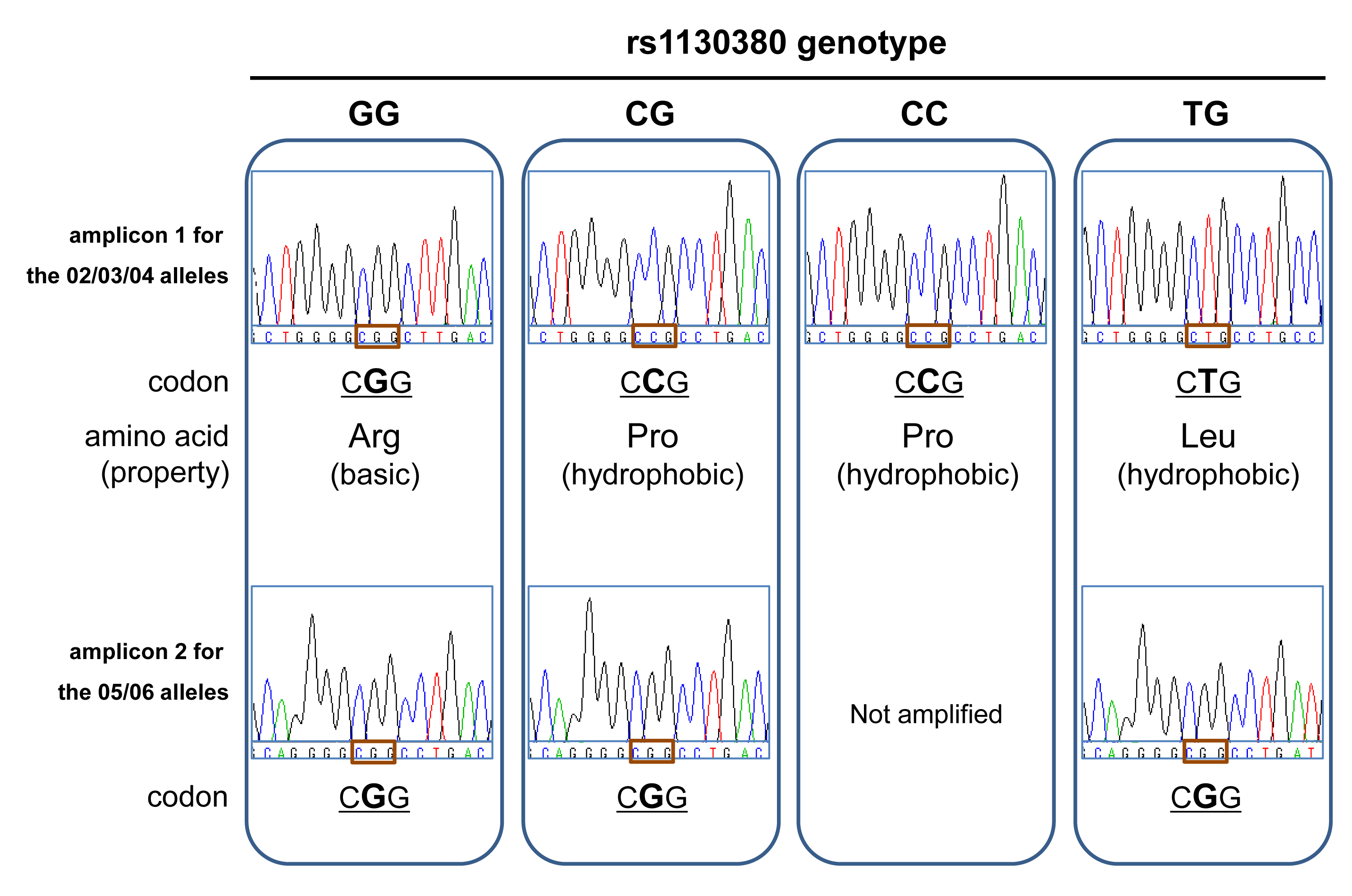

Supplement: Figure S6 — Chromatograms of different alleles of rs1130380 and their predicted effects on protein translation. Two different PCR amplifications were performed for genotyping of HLA-DQB1 alleles: amplicon 1 for the 02/03/04 alleles and amplicon 2 for the 05/06 alleles [33]. The G to C nucleotide change of codon 55 leads to amino acid substitution from Arg to Pro. The properties of Arg and Pro are basic and hydrophobic, respectively. We also observed the G to T nucleotide change, but this variant was not considered for further analysis because of its low frequency (< 1%). (TIF) [file pone.0084226.s006.tif]

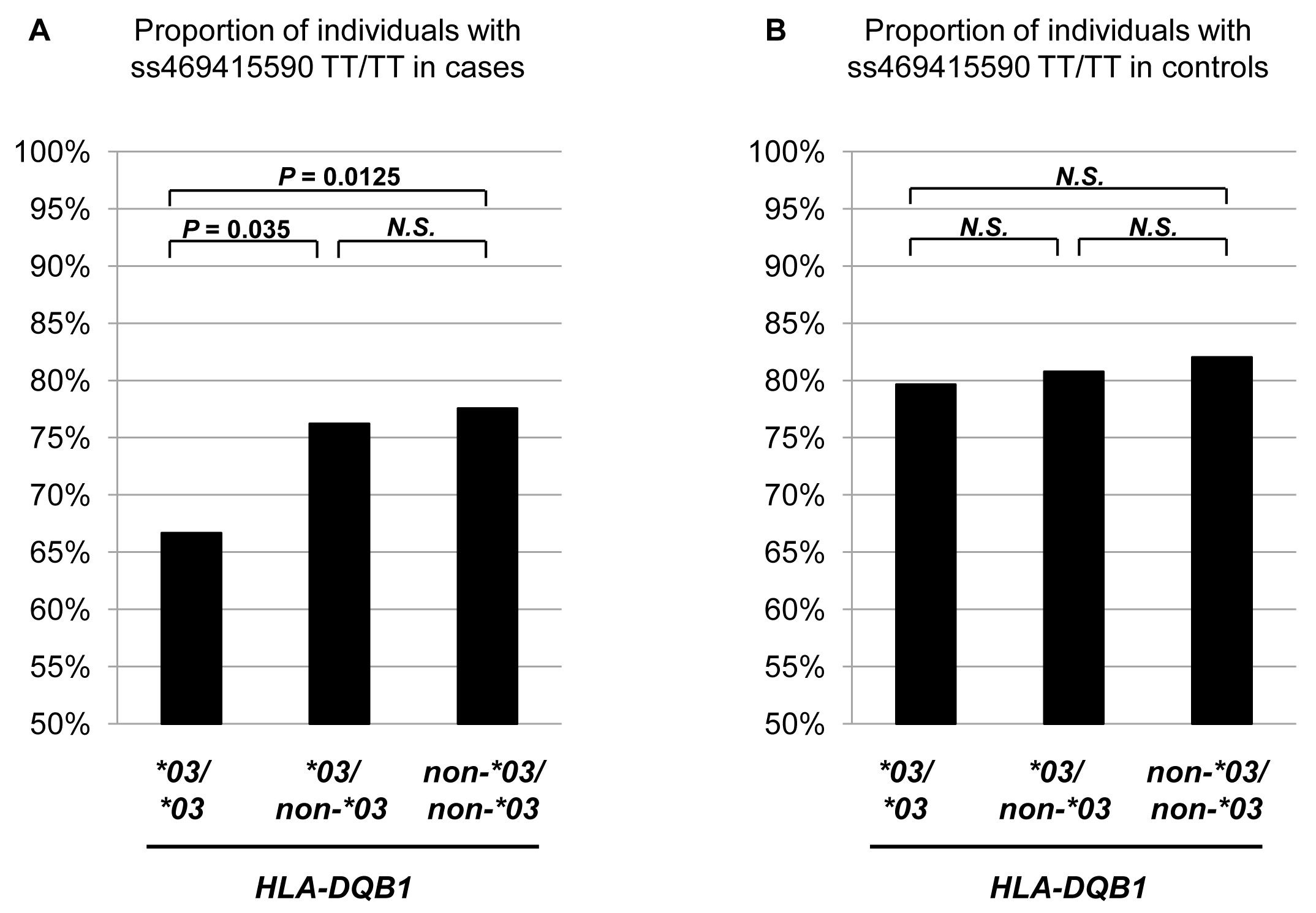

Supplement: Figure S7 — Relationship between HLA-DQB1*03 (rs1130380) and IFNL4 variant (ss469415590) in chronic HCV patients (A) and healthy controls (B). Proportion of individuals with ss469415590 TT/TT in cases and controls according to HLA-DQB1*03 status. P values were calculated using the chi-squared test. (TIF) [file pone.0084226.s007.tif]
