# Supplementary material for: HLA-DQB1*03 Confers Susceptibility to Chronic Hepatitis C in Japanese: A Genome-Wide Association Study
Source: PLoS One. 2013 Dec 20;8(12):e84226. doi: 10.1371/journal.pone.0084226 (PMC3871580; doi:10.1371/journal.pone.0084226)
Supplement: Table S1 — Results of GWAS. (PDF) [file pone.0084226.s008.pdf]

Table S1. Results of GWAS.

SNP	Chr	Position	Allele [1/2]	Case 11	Case 12	Case 22	Control 11	Control 12	Control 22	MAF Case	MAF Control	OR[a]	(95%CI)	*P*[b]	Nearest gene
rs6933400	6	31015155	TC	11	123	347	29	485	2449	0.151	0.092	0.57	(0.47-0.69)	1.92E-08	*LOC100129065*
rs6911476	6	26720342	AG	4	88	389	7	328	2625	0.100	0.058	0.55	(0.44-0.7)	6.11E-07	*ABT1*
rs7122630	11	20913841	TC	476	5	0	2961	1	0	0.005	0.000	30.95	(3.61-265.18)	9.33E-07	*NELL1*
rs2523477	6	31468368	TC	377	100	4	2583	359	20	0.112	0.067	1.75	(1.4-2.19)	9.98E-07	*LOC100129668*
rs2523476	6	31469403	TC	4	100	377	20	359	2583	0.112	0.067	0.57	(0.46-0.71)	9.98E-07	*LOC100129668*
rs7356849	6	26504718	AG	384	92	5	2597	355	11	0.106	0.064	1.75	(1.39-2.2)	1.56E-06	*BTN2A2*
rs2596551	6	31440218	AC	374	101	6	2557	376	25	0.117	0.072	1.72	(1.38-2.14)	1.83E-06	*LOC729816*
rs11753872	6	29844921	TG	5	103	373	21	388	2548	0.117	0.073	0.59	(0.47-0.73)	2.24E-06	*HCP5P14*
rs9275572	6	32786977	TC	96	217	168	366	1317	1280	0.425	0.346	0.71	(0.62-0.82)	2.62E-06	*HLA-DQA2*
rs1883214	6	26414663	AC	7	113	361	17	474	2472	0.132	0.086	0.62	(0.5-0.76)	3.38E-06	*TRNAS-GCU*
rs7651342	3	82582297	TC	386	89	6	2076	800	85	0.105	0.164	0.60	(0.48-0.74)	3.69E-06	*CYP51P1*
rs11758087	6	29872422	TC	5	103	373	21	394	2548	0.117	0.074	0.60	(0.48-0.74)	3.75E-06	*HCG4P9*
rs17475879	6	30472487	AG	5	100	376	16	390	2547	0.114	0.071	0.60	(0.48-0.74)	4.00E-06	*LOC100129192*
rs9261588	6	30318786	AC	6	99	376	17	394	2552	0.115	0.072	0.60	(0.48-0.74)	4.11E-06	*HLA-L*
rs6041027	20	11926265	TC	142	251	88	682	1455	825	0.444	0.476	0.72	(0.63-0.83)	4.21E-06	*BTBD3*
rs3094065	6	30390311	AG	6	99	376	17	394	2550	0.115	0.072	0.60	(0.48-0.74)	4.24E-06	*LOC100133303*
rs3846382	4	19219037	AG	109	224	148	888	1421	652	0.459	0.460	1.38	(1.2-1.58)	5.61E-06	*LOC645174*
rs6456730	6	26592355	TC	389	88	4	2612	342	9	0.100	0.061	1.71	(1.35-2.17)	5.63E-06	*BTN2A1*
rs10934619	3	124049863	AG	214	211	56	1017	1445	501	0.336	0.413	0.72	(0.62-0.83)	5.97E-06	*DIRC2*
rs1488839	4	19326552	TC	107	227	147	873	1436	654	0.458	0.463	1.37	(1.19-1.57)	8.39E-06	*LOC645174*
rs3734526	6	25887715	AC	397	80	4	2642	311	7	0.091	0.055	1.73	(1.36-2.22)	8.74E-06	*SLC17A4*
rs9275563	6	32785890	AG	93	208	180	720	1448	791	0.410	0.488	1.37	(1.2-1.58)	8.99E-06	*HLA-DQA2*
rs3130361	6	30447543	AG	7	102	372	20	420	2523	0.121	0.078	0.61	(0.49-0.76)	9.00E-06	*RPP21*
rs3757333	6	30136585	TG	5	104	372	23	403	2534	0.119	0.076	0.61	(0.49-0.76)	9.21E-06	*ZNRD1*
rs2508015	6	31118179	TC	5	114	362	26	449	2488	0.129	0.085	0.62	(0.51-0.77)	9.78E-06	*LOC729792*

MAF; minor allele frequency, Chr; Chromosome, OR; odds ratio, CI; confidence interval.

[a]Odds ratio of allele[1] as reference. [b]*P* value of Cochran-Armitage trend test.
