# Supplementary material for: HLA-DQB1*03 Confers Susceptibility to Chronic Hepatitis C in Japanese: A Genome-Wide Association Study
Source: PLoS One. 2013 Dec 20;8(12):e84226. doi: 10.1371/journal.pone.0084226 (PMC3871580; doi:10.1371/journal.pone.0084226)
Supplement: Table S2 — Results of 1st replication study. (PDF) [file pone.0084226.s009.pdf]

Table S2. Results of 1st replication study.

SNP	Chr	Position	Allele [1/2]	Case 11	Case 12	Case 22	Control 11	Control 12	Control 22	MAF Case	MAF Control	OR[a]	(95%CI)	*P*[b]
rs6933400	6	31015155	TC	34	720	3602	6	183	924	0.090	0.088	0.97	(0.82-1.14)	6.73E-01
rs6911476	6	26720342	AG	15	433	3909	3	105	1006	0.053	0.050	0.93	(0.76-1.16)	5.34E-01
rs7122630	11	20913841	TC	4347	9	0	1108	6	0	0.001	0.003	0.38	(0.14-1.08)	5.86E-02
rs2523476	6	31469403	TC	34	482	3838	6	112	995	0.063	0.056	0.88	(0.72-1.07)	2.05E-01
rs2596551	6	31440218	AC	3813	503	27	992	117	5	0.064	0.057	1.13	(0.93-1.38)	2.23E-01
rs11753872	6	29844921	TG	24	548	3780	6	134	974	0.068	0.066	0.95	(0.79-1.15)	6.24E-01
rs9275572	6	32786977	TC	634	2049	1673	119	474	519	0.381	0.320	0.77	(0.69-0.85)	1.40E-07
rs1883214	6	26414663	AC	34	667	3656	6	156	952	0.084	0.075	0.89	(0.74-1.05)	1.72E-01
rs7651342	3	82582297	TC	3110	1134	114	785	302	27	0.156	0.160	0.97	(0.86-1.11)	6.85E-01
rs17475879	6	30472487	AG	21	542	3793	7	125	982	0.067	0.062	0.93	(0.76-1.12)	4.34E-01
rs9261588	6	30318786	AC	22	520	3813	5	128	981	0.065	0.062	0.95	(0.79-1.16)	6.31E-01
rs6041027	20	11926265	TC	1002	2153	1192	255	537	320	0.478	0.471	0.97	(0.88-1.07)	5.37E-01
rs3846382	4	19219037	AG	1187	2179	990	295	538	274	0.477	0.491	0.95	(0.86-1.04)	2.70E-01
rs10934619	3	124049863	AG	1555	2110	681	384	552	177	0.399	0.407	0.97	(0.88-1.07)	5.13E-01
rs1488839	4	19326552	TC	1194	2156	1003	286	564	263	0.478	0.490	0.95	(0.87-1.05)	3.29E-01
rs3734526	6	25887715	AC	3916	389	12	1013	96	1	0.048	0.044	1.09	(0.87-1.36)	4.65E-01
rs3757333	6	30136585	TG	29	553	3770	6	132	975	0.070	0.065	0.92	(0.76-1.11)	3.67E-01

MAF; minor allele frequency, Chr; Chromosome, OR; odds ratio, CI; confidence interval.

[a]Odds ratio of allele[1] as reference. [b]*P* value of Cochran-Armitage trend test.
