# Supplementary material for: HLA-DQB1*03 Confers Susceptibility to Chronic Hepatitis C in Japanese: A Genome-Wide Association Study
Source: PLoS One. 2013 Dec 20;8(12):e84226. doi: 10.1371/journal.pone.0084226 (PMC3871580; doi:10.1371/journal.pone.0084226)
Supplement: Table S3 — Multiple logistic regression analysis for the risk of chronic hepatitis C using 1st replication samples (4,347 cases and 1,097 controls). (PDF) [file pone.0084226.s010.pdf]

Table S3. Multiple logistic regression analysis for the risk of chronic hepatitis C using 1st replication samples (4,347 cases and 1,097 controls).

	OR	(95%CI)	***P***
rs9275572 (C allele)	0.79	(0.70-0.89)	1.43E-04
Male gender	2.04	(1.73-2.40)	< 2E-16
Age (y)	1.10	(1.10-1.11)	< 2E-16

OR; odds ratio, CI; confidence interval.
