# Supplementary material for: HLA-DQB1*03 Confers Susceptibility to Chronic Hepatitis C in Japanese: A Genome-Wide Association Study
Source: PLoS One. 2013 Dec 20;8(12):e84226. doi: 10.1371/journal.pone.0084226 (PMC3871580; doi:10.1371/journal.pone.0084226)
Supplement: Table S4 — (A) Effect of rs9275572 on the susceptibility of chronic hepatitis C stratified by gender and age using 1st replication samples. (B) Effect of rs9275572 on the susceptibility of chronic hepatitis C stratified by HCV genotype using 1st replication samples. (PDF) [file pone.0084226.s011.pdf]

Table S4A. Effect of rs9275572 on the susceptibility of chronic hepatitis C stratified by gender and age using 1st replication samples.

Subgroup	Case			Control			Protective AF		OR (95%CI)[a]	P^{b}	P_{het}^{c}
	TT	TC	CC	TT	TC	CC	Case	Control			
Male	318	1107	941	46	177	191	0.632	0.675	0.83 (0.71-0.97)	1.63E-02	0.151
Female	317	945	735	73	297	328	0.605	0.683	0.71 (0.62-0.81)	2.77E-07	
Age=<40	46	131	117	63	264	282	0.621	0.680	0.77 (0.63-0.95)	1.41E-02	0.930
40<Age=<55	139	426	345	32	102	123	0.613	0.677	0.76 (0.61-0.93)	9.27E-03	
55<Age	444	1488	1211	24	103	104	0.622	0.673	0.80 (0.65-0.98)	2.79E-02	

AF; allele frequency, OR; odds ratio, CI; confidence interval.

[a]Odds ratios of protective allele (C) from two-by-two allele frequency table. [b]*P* value of Cochran-Armitage trend test. [c]Result of Breslow-Day test.

Table S4B. Effect of rs9275572 on the susceptibility of chronic hepatitis C stratified by HCV genotype using 1st replication samples.

		vs Control (n=1,112, Protective AF = 0.680)		
	Protective AF	OR (95%CI)[a]	P^{b}	P_{het}^{c}
HCV genotype 1 (n=2,890)	0.610	0.74 (0.66-0.82)	7.88E-09	0.127
HCV genotype 2 (n=1,268)	0.639	0.83 (0.74-0.94)	3.52E-03	

AF; allele frequency, OR; odds ratio, CI; confidence interval.

[a]Odds ratios of protective allele (C) from two-by-two allele frequency table. [b]*P* value of Cochran-Armitage trend test. [c]Result of Breslow-Day test.
