# Supplementary material for: HLA-DQB1*03 Confers Susceptibility to Chronic Hepatitis C in Japanese: A Genome-Wide Association Study
Source: PLoS One. 2013 Dec 20;8(12):e84226. doi: 10.1371/journal.pone.0084226 (PMC3871580; doi:10.1371/journal.pone.0084226)
Supplement: Table S5 — (A) Useful variants for genotyping common HLA-DQA1 haplotypes in the Japanese population. (B) Useful variants for genotyping common HLA-DQB1 haplotypes in the Japanese population. (PDF) [file pone.0084226.s012.pdf]

Table S5A. Useful variants for genotyping common *HLA-DQA1* haplotypes in the Japanese population.

Variation	rs12722051	rs10093	rs1142324	rs9272709
Location	32,549,147	32,549,173	32,549,207	32,549,279
*0101	A	G	C	C
*0102	A	C	C	C
*0103	T	C	C	C
*0301	A	G	T	T
*0401	A	C	T	C
*0501	A	C	T	T
*0601	T	C	T	C

Table S5B. Useful variants for genotyping common *HLA-DQB1* haplotypes in the Japanese population.

Variation	rs41540813	rs1130370	rs1063318	rs1049083	rs1140313	rs1130380	rs41552812	rs1071637
Location	32,572,790	32,572,749	32,572,745	32,572,724	32,572,700	32,572,694	32,572,689	32,572,688
*0301	G	T	C	A	T	C	G	A
*0302	G	T	C	G	T	C	G	C
*0303	G	T	C	G	T	C	G	A
*0401	T	T	C	G	T	G	G	A
*0402	G	T	C	G	T	G	G	A
*0501	G	T	T	G	A	G	G	T
*0502	G	T	T	G	A	G	A	G
*0503	G	T	T	G	A	G	G	A
*0601	G	G	T	G	A	G	G	A
*0602	G	T	C	G	A	G	G	A
*0604	G	T	C	G	A	G	G	T
