# Supplementary material for: HLA-DQB1*03 Confers Susceptibility to Chronic Hepatitis C in Japanese: A Genome-Wide Association Study
Source: PLoS One. 2013 Dec 20;8(12):e84226. doi: 10.1371/journal.pone.0084226 (PMC3871580; doi:10.1371/journal.pone.0084226)
Supplement: Table S6 — Haplotype analysis was performed using the landmark SNP (rs9275572) and 12 variants shown in Table S5A and S5B by HaploView software. (PDF) [file pone.0084226.s013.pdf]

Table S6. Haplotype analysis.

Haplotype			Frequency				
HLA-DQA1	HLA-DQB1	rs9275572	Case	Control	OR[a]	(95%CI)	*P*[b]
*0103	*0601	C	0.210	0.189	1.15	(0.98-1.34)	0.083
*0301	*0303	C	0.130	0.175	0.70	(0.59-0.83)	5.68E-05
*0301	*0401	T	0.140	0.133	1.05	(0.88-1.26)	0.558
*0301	*0302	C	0.093	0.102	0.90	(0.73-1.11)	0.317
*0101	*0501	C	0.070	0.079	0.88	(0.70-1.11)	0.282
*0102	*0602	T	0.073	0.060	1.22	(0.95-1.56)	0.119
*0501	*0301	C	0.060	0.069	0.87	(0.67-1.11)	0.262
*0102	*0604	T	0.057	0.047	1.25	(0.94-1.64)	0.122
*0101	*0503	T	0.054	0.036	1.54	(1.13-2.08)	0.005
*0301	*0402	T	0.029	0.019	1.54	(1.02-2.32)	0.040
*0101	*0502	C	0.023	0.023	1.00	(0.66-1.51)	0.997
*0401	*0402	T	0.024	0.019	1.30	(0.85-1.98)	0.225
*0601	*0301	C	0.013	0.021	0.64	(0.39-1.04)	0.070

[a]Odds ratios of haplotype from two-by-two frequency table. [b]*P* value of chi-squared test.

Haplotype analysis was performed using landmark SNP (rs9275572) and 12 variants shown in Table S5A and S5B by HaploView software.

Only haplotypes with frequencies of more than 1% were shown.
