# Supplementary material for: HLA-DQB1*03 Confers Susceptibility to Chronic Hepatitis C in Japanese: A Genome-Wide Association Study
Source: PLoS One. 2013 Dec 20;8(12):e84226. doi: 10.1371/journal.pone.0084226 (PMC3871580; doi:10.1371/journal.pone.0084226)
Supplement: Table S7 — Single marker effect on chronic hepatitis C. (PDF) [file pone.0084226.s014.pdf]

Table S7. Single marker effect on chronic hepatitis C.

	Single marker	Protective allele	Frequency		OR[a]	(95%CI)	*P*[b]
			Case	Control			
DQA1	rs12722051	A	0.776	0.789	0.92	(0.79-1.07)	2.80E-01
DQA1	rs10093	G	0.543	0.571	0.89	(0.79-1.01)	7.14E-02
DQA1	rs1142324	T	0.506	0.558	0.81	(0.72-0.92)	9.00E-04
DQA1	rs9272709	T	0.460	0.508	0.83	(0.73-0.94)	2.40E-03
DQB1	rs1071637	C and G	0.259	0.270	0.94	(0.82-1.08)	4.14E-01
DQB1	rs41552812	A	0.029	0.029	0.98	(0.68-1.41)	9.08E-01
DQB1	rs1130380	C	0.313	0.387	0.72	(0.63-0.82)	6.08E-07
DQB1	rs1140313	T	0.507	0.561	0.81	(0.71-0.91)	5.00E-04
DQB1	rs1049083	A	0.083	0.096	0.85	(0.68-1.05)	1.32E-01
DQB1	rs1063318	C	0.637	0.668	0.87	(0.77-0.99)	3.63E-02
DQB1	rs1130370	T	0.790	0.812	0.87	(0.75-1.02)	8.50E-02
DQB1	rs41540813	G	0.860	0.866	0.95	(0.79-1.13)	5.39E-01
Landmark SNP	rs9275572	C	0.623	0.686	0.76	(0.66-0.86)	2.07E-05

[a]Odds ratios of protective allele from two-by-two allele table. [b]*P* value of chi-squared test.
