# Supplementary material for: HLA-DQB1*03 Confers Susceptibility to Chronic Hepatitis C in Japanese: A Genome-Wide Association Study
Source: PLoS One. 2013 Dec 20;8(12):e84226. doi: 10.1371/journal.pone.0084226 (PMC3871580; doi:10.1371/journal.pone.0084226)
Supplement: Table S8 — Results of GWAS of two SNPs. (PDF) [file pone.0084226.s015.pdf]

Table S8. Results of GWAS of two SNPs.

		Allele	Case			Control			MAF				GWAS
Locus	**SNP**	**[1/2]**	**11**	**12**	**22**	**11**	**12**	**22**	**Case**	**Control**	**OR[a] (95%CI)**	***P*[b]**	**ranking**
HLA-DQ	rs9275572	C/T	96	217	168	366	1317	1280	0.425	0.346	0.71 (0.62-0.82)	2.62E-06	9
IL28B	rs8099917	G/T	12	104	365	37	525	2401	0.133	0.101	0.73 (0.60-0.90)	3.22E-03	1640

MAF; minor allele frequency, OR; odds ratio, CI; confidence interval.

[a]Odds ratio of allele[1] as reference. [b]*P* value of Cochran-Armitage trend test.
