# Supplementary material for: HLA-DQB1*03 Confers Susceptibility to Chronic Hepatitis C in Japanese: A Genome-Wide Association Study
Source: PLoS One. 2013 Dec 20;8(12):e84226. doi: 10.1371/journal.pone.0084226 (PMC3871580; doi:10.1371/journal.pone.0084226)
Supplement: Table S9 — Case-control association analysis of 5 variants under different genetic models. (PDF) [file pone.0084226.s016.pdf]

Table S9. Case-control association analysis of 5 variants under different genetic models

			Genetic Model					
	Protective AF		Genotype [a]	Allelic [b]	Dominant [c]		Recessive [c]	
SNP	Case	Control	*P*	*P*	*P*	OR (95%CI)	*P*	OR (95%CI)
rs8099917	0.877	0.903	2.07E-02	5.65E-03	3.16E-01	0.66 (0.30-1.49)	5.95E-03	0.75 (0.60-0.92)
rs12979860	0.872	0.899	1.52E-02	4.19E-03	3.34E-01	0.68 (0.32-1.48)	4.10E-03	0.74 (0.60-0.91)
ss469415590	0.872	0.900	1.12E-02	3.03E-03	3.34E-01	0.68 (0.32-1.48)	2.88E-03	0.73 (0.59-0.90)
rs9275572	0.616	0.682	5.30E-05	7.26E-06	5.34E-04	0.64 (0.49-0.82)	1.63E-04	0.72 (0.61-0.85)
rs1130380	0.313	0.383	1.98E-05	1.62E-06	2.22E-05	0.69 (0.58-0.82)	1.03E-03	0.66 (0.51-0.85)

AF; allele frequency, OR; odds ratio, CI; confidence interval. *P* values were calculated using chi-squared test.

[a] the test of association in the 2-by-3 table of disease-by-genotype. [b] the test of association under the allelic model.

[c] the test of association under the dominant and recessive models for the protective allele.
