# Supplementary material for: HLA-DQB1*03 Confers Susceptibility to Chronic Hepatitis C in Japanese: A Genome-Wide Association Study
Source: PLoS One. 2013 Dec 20;8(12):e84226. doi: 10.1371/journal.pone.0084226 (PMC3871580; doi:10.1371/journal.pone.0084226)
Supplement: Table S10 — Basic characteristics of study population. (PDF) [file pone.0084226.s017.pdf]

Table S10. Basic characteristics of study population.

	Case	Control
**GWAS**		
n	481	2,963
Source	Toranomon Hosp.	BioBank Japan[a] (n=2,057)
		Healthy volunteer (Osaka-Midosuji Rotary Club) (n=906)
Platform	Illumina HumanHap610k	Illumina HumanHap550k
Male ratio	0.64	0.533
mean age (sd)	56.4 (10.3)	51.9 (16.3)
HCV genotype 1 / 2 / 3	481 / 0 / 0	
**1st replication**		
n	4,358	1,114
Source	Hiroshima Univ.	Healthy volunteer (Hiroshima Univ.)
Platform	Invader assay	Invader assay
Male ratio	0.542	0.373
mean age (sd)	61.2 (12.1)	41.3 (15.0)
HCV genotype 1 / 2 / 3	2,890 / 1,268 / 9	
1+2 / not tested	12 / 183	
**2nd replication**		
n	1,379	25,817
Source	Toranomon Hosp. (1,004)	BioBank Japan[a]
	SapporoKosei Hosp. (375)	
Platform	Invader assay	Illumina HumanHap610k
Male ratio	0.513	0.519
mean age (sd)	59.1 (11.5)	63.1 (12.0)
HCV genotype 1 / 2 / 3	1,066 / 313 / 0	

[a]The control groups from BioBank Japan consisted of individuals with diseases unrelated to chronic liver diseases.
